# Association of metabolic score for insulin resistance with gestational diabetes mellitus: a multicenter cohort study

**DOI:** 10.3389/fnut.2025.1661119

**Published:** 2025-09-24

**Authors:** Qiong Li, Ailing Chen, Chenyang Zhao, Ying Zhang, Meng Li, Ying Gu, Yajing Pang, Ping Yu, Chaoyan Yue

**Affiliations:** ^1^Department of Obstetrics and Gynecology, The First People’s Hospital of Chenzhou, Chenzhou, China; ^2^Department of Obstetrics and Gynecology, Women’s Hospital of Jiangnan University, Wuxi Maternity and Child Health Care Hospital, Wuxi, China; ^3^Obstetrics & Gynecology Hospital of Fudan University, Shanghai Key Lab of Reproduction and Development, Shanghai Key Lab of Female Reproductive Endocrine Related Diseases, Shanghai, China; ^4^Wuxi School of Medicine, Jiangnan University, Wuxi, Jiangsu, China; ^5^Center of Reproductive Medicine, Women’s Hospital of Jiangnan University, Wuxi Maternity and Child Health Care Hospital, Wuxi, China

**Keywords:** METS-IR, insulin resistance, gestational diabetes mellitus, cohort study, multicenter

## Abstract

**Background:**

The metabolic score for insulin resistance (METS-IR) is a novel and effective indicator for assessing insulin resistance. Previous studies have shown that METS-IR is positively associated with the risk of type 2 diabetes. However, the association between METS-IR and gestational diabetes mellitus (GDM) has not yet been clearly clarified. This study aims to investigate the association between METS-IR and GDM as well as its related adverse pregnancy outcomes and to evaluate its predictive value.

**Methods:**

A total of 37,770 singleton pregnant women from three hospitals in China between January 2018 and June 2024 were included in the study. METS-IR was calculated using the formula: ln ([high-density lipoprotein cholesterol (HDL-C) (mg/dL)] × [2 × fasting glucose (mg/dL)] + TG (mg/dL) × BMI (kg/m^2^)). Participants were divided into four groups according to METS-IR quartiles. Multivariable logistic regression models, smoothed curve fitting, and subgroup analyses were conducted to assess the associations between METS-IR and GDM as well as related adverse pregnancy outcomes. The receiver operating characteristic (ROC) curves were used to evaluate the predictive performance.

**Results:**

After adjusting for potential confounders, higher METS-IR levels were significantly associated with an increased risk of GDM. Compared with the lowest quartile group (Q1), the risks of GDM in the Q2, Q3, and Q4 groups increased by 13% (OR = 1.13, 95% CI: 1.02–1.25), 59% (OR = 1.59, 95% CI: 1.44–1.75), and 165% (OR = 2.65, 95% CI: 2.42–2.91), respectively. Similar associations were also observed between METS-IR and preterm birth, macrosomia, gestational diabetes mellitus (GDM) complicated with preeclampsia (GDM&PE), and pharmacologically treated GDM class A2 (GDMA2). Smoothed curve fitting suggested an approximately linear dose–response relationship between METS-IR and GDM. Subgroup analysis indicated that the association between METS-IR and GDM remained consistent across different age groups (interaction *p* > 0.05), with a higher GDM risk observed among women aged ≥35 years. The ROC analysis showed that the areas under the curve (AUCs) of METS-IR for predicting GDM, preterm birth, macrosomia, GDM&PE, and GDMA2 were 0.623, 0.532, 0.640, 0.741, and 0.712, respectively.

**Conclusion:**

This study demonstrated that METS-IR is positively associated with GDM risk and its related adverse pregnancy outcomes. METS-IR may serve as a useful tool for risk stratification and early intervention in clinical practice for GDM.

## Introduction

Gestational diabetes mellitus (GDM) refers to abnormal glucose tolerance first detected during pregnancy and is one of the most common metabolic complications encountered in pregnant women (1). Globally, the prevalence of GDM is approximately 16.7% (2). In recent years, with increasing obesity rates and a rising proportion of advanced maternal age pregnancies, the incidence of GDM has continued to increase, posing significant threats to both maternal and fetal health (3). GDM is closely associated with multiple adverse pregnancy outcomes, including hypertensive disorders of pregnancy, preterm birth, and macrosomia (4). It also significantly increases the long-term risk of type 2 diabetes in mothers, as well as the likelihood of obesity and metabolic syndrome in offspring (5, 6). Existing evidence suggests that women who develop GDM exhibit enhanced insulin resistance (IR) and impaired β-cell compensation early in pregnancy (7), indicating that metabolic disturbances may occur before clinical diagnosis. Therefore, applying effective metabolic evaluation tools for risk stratification of GDM and timely intervention is important for improving pregnancy outcomes and interrupting the intergenerational transmission of metabolic disorders.

IR is one of the key pathophysiological mechanisms underlying the development of GDM and serves as an important marker for predicting and assessing GDM risk (8, 9). However, traditional methods for evaluating IR, such as the euglycemic-hyperinsulinemic clamp (EHC), although considered the gold standard, are technically complex and invasive, which limits their applicability in routine clinical screening (10). Thus, there is a pressing need to develop simple, non-invasive, and accurate surrogate markers for IR to improve early risk stratification of GDM. The metabolic score for insulin resistance (METS-IR), developed by the team of Professor Bello-Chavolla OY, is a novel cardiometabolic risk scoring model. This score incorporates routinely available clinical parameters, including glucose-related indices (fasting plasma glucose, FPG), lipid profiles (such as triglycerides (TG) and high-density lipoprotein cholesterol (HDL-C)), and obesity-related measures (such as body mass index (BMI)) (11). Studies have demonstrated that METS-IR outperforms EHC in detecting impaired insulin sensitivity (11), with good reproducibility and ease of calculation, making it a promising indirect tool for assessing IR. Some cohort studies have consistently shown a positive association between METS-IR and the risk of developing type 2 diabetes (12–15). Recent studies have demonstrated an association between elevated METS-IR and an increased risk of GDM. One study using NHANES data included 5,189 pregnant women (417 with GDM) and found a significant association between higher METS-IR and GDM, particularly among women with high school education or higher (16). Another cohort study in Iran (*n* = 1,845) reported that first-trimester METS-IR may predict GDM in Iranian women (17). While these studies support the potential of METS-IR as a predictive tool, they have limitations, including single-center design, small sample size, and reliance on self-reported data, which may affect generalizability and accuracy. To address these limitations, our multicenter study with a large sample size rigorously controlled for confounders and applied multiple statistical methods to examine the association between METS-IR and both GDM and related adverse pregnancy outcomes. Our findings aim to provide a simple, practical clinical indicator for early risk stratification and prediction of GDM.

## Methods

### Study design

**Figure 1:**
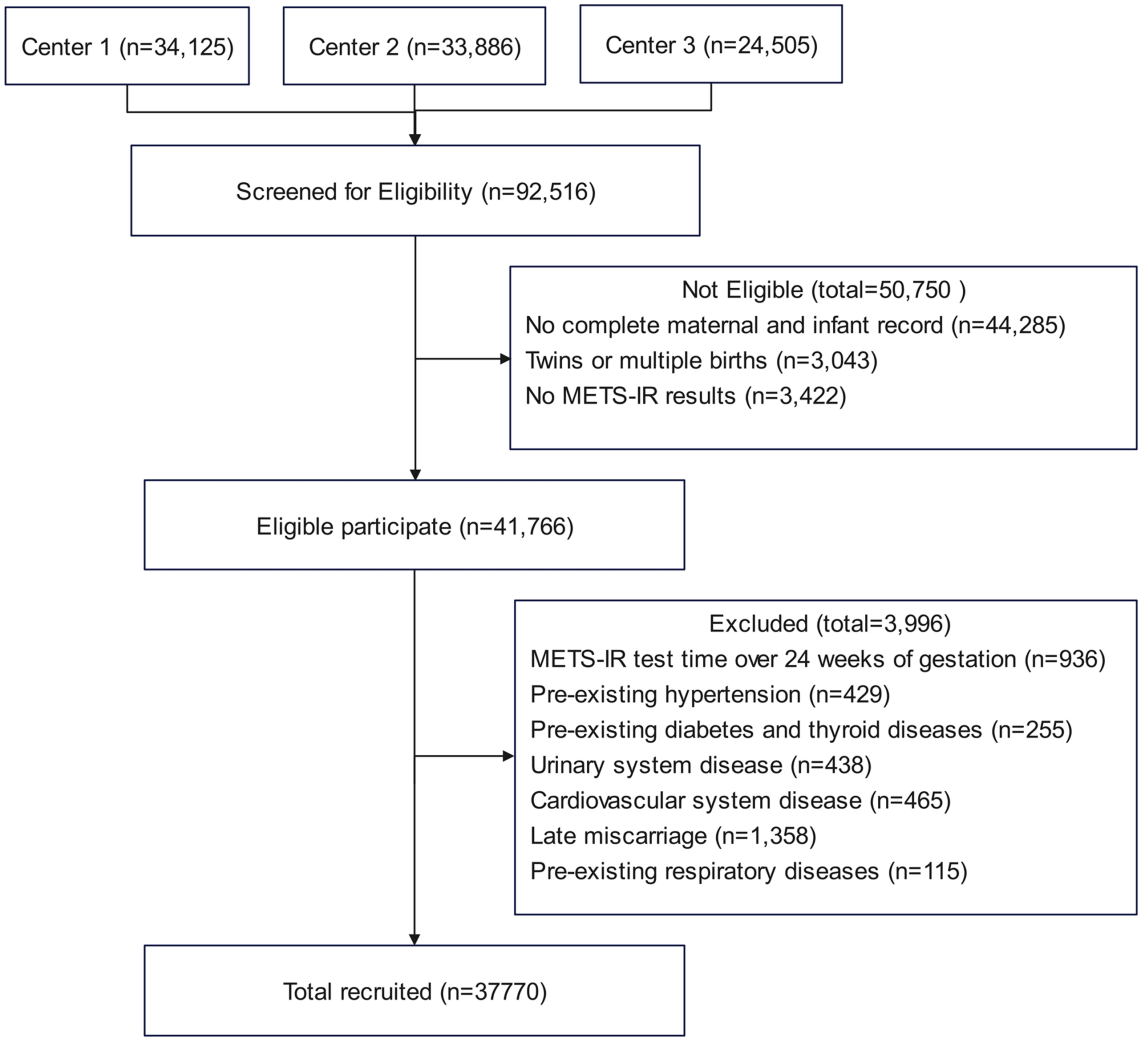
Flowchart of study participants.

This study is a multicenter retrospective cohort study conducted between January 2018 and June 2024. A total of 37,770 singleton pregnant women were enrolled from three medical centers: Obstetrics and Gynecology Hospital of Fudan University, Huangpu Branch (Center 1), Obstetrics and Gynecology Hospital of Fudan University, Yangpu Branch (Center 2), and the First People’s Hospital of Chenzhou (Center 3). Among them, 5,166 were diagnosed with GDM, and 32,604 had normal glucose tolerance. Baseline clinical data and laboratory tests were collected at the first antenatal visit and obtained from the Hospital Information System (HIS) and Laboratory Information System (LIS). The inclusion criteria were as follows: (1) singleton pregnancy and (2) delivery at any of the participating hospitals. The exclusion criteria were as follows: initial measurements of FPG, TG, or HDL-C after 24 weeks of gestation, multiple pregnancies, preexisting diabetes or other endocrine disorders, chronic hypertension, cardiovascular disease, renal disease, or respiratory disease, and incomplete maternal or neonatal records. Figure 1 illustrates the participant inclusion process. The study was approved by the Ethics Committees of the Obstetrics and Gynecology Hospital of Fudan University and the First People’s Hospital of Chenzhou. All participants provided broad informed consent, and the study was conducted in accordance with the principles of the Declaration of Helsinki.

### Variables and measurements

The primary exposure variable was METS-IR. Demographic and clinical data, including age, prepregnancy body mass index (BMI), history of chronic diseases (hypertension, diabetes, etc.), and education level, were collected by trained healthcare professionals. Laboratory data were obtained from the first antenatal visit before 24 weeks of gestation. After an overnight fast of at least 8 h, venous blood samples were drawn and analyzed using an automatic biochemical analyzer to measure FPG (mmol/L), TG (mmol/L), HDL-C (mmol/L), alanine aminotransferase (ALT, U/L), creatinine (Cr, μmol/L), and total cholesterol (TC, mmol/L). METS-IR was calculated as follows: ln[(2 × FPG (mg/dL) + TG (mg/dL)) × BMI (kg/m^2^)]/ln[HDL-C (mg/dL)], where BMI was calculated as prepregnancy weight (kg) divided by height squared (m^2^) (11). The METS-IR in this study ranged from 21.58 to 58.43 and was categorized into four quartiles: Q1 (<32.18), Q2 (32.18–36.10), Q3 (36.10–40.91), and Q4 (>40.91). Maternal and neonatal clinical data were collected postpartum, mainly including gestational age at delivery and neonatal birth weight.

Based on previous literature and clinical expertise, potential confounding variables were selected, including age, test week, Cr, ALT, TC, history of hypertension, history of diabetes, tobacco use, alcohol consumption, *in vitro* fertilization (IVF), adverse pregnancy history, parity, and education level. Age was stratified into <35 years and ≥35 years according to WHO guidelines (18). Adverse pregnancy history was defined as prior spontaneous abortion or major obstetric complications. Education levels were categorized into postgraduate or above, bachelor’s degree, associate degree, senior high school, and junior high school or below.

### Outcomes and measurements

The primary outcome was GDM. Diagnosis was based on the International Association of Diabetes and Pregnancy Study Groups (IADPSG) criteria using a 75-g oral glucose tolerance test (OGTT) performed between 24 and 28 weeks of gestation. GDM was diagnosed if any one of the following thresholds was met: fasting glucose ≥5.1 mmol/L, 1-h glucose ≥ 10.0 mmol/L, or 2-h glucose ≥ 8.5 mmol/L (19).

Secondary outcomes included preterm birth, macrosomia, GDM complicated with preeclampsia (GDM&PE), and pharmacologically treated GDM (GDMA2). Preterm birth was defined as delivery before 37 weeks of gestation (20). Macrosomia was defined as a birth weight ≥ 4,000 g (21). GDM&PE was defined as the coexistence of GDM and preeclampsia diagnosed after 20 weeks of gestation. Preeclampsia was diagnosed according to the 2020 American College of Obstetricians and Gynecologists (ACOG) (22). For women with regular menstrual cycles, fetal gestational age was estimated based on the last menstrual period. For those with irregular cycles, early ultrasound findings were used for gestational dating.

### Statistical analysis

Baseline characteristics of the study population were summarized across METS-IR quartiles (Q1–Q4). Continuous variables with normal distribution were expressed as mean ± standard deviation (SD), while skewed variables were presented as median (interquartile range, IQR). Categorical variables were described as frequency and percentage (%).

METS-IR was categorized into quartiles and used as a categorical variable, with the lowest quartile (Q1) serving as the reference group. Multivariable logistic regression models were applied to assess the associations between METS-IR and both the primary and secondary outcomes. The results were presented as odds ratios (ORs) with 95% confidence intervals (CIs). According to the STROBE statement (23), two models were constructed: Model I was unadjusted, and Model II was adjusted for age, test week, Cr, ALT, TC, history of hypertension, history of diabetes, tobacco use, alcohol consumption, IVF, adverse pregnancy history, parity, and education levels.

To explore potential effect modification, subgroup analyses were conducted by age, and multivariable logistic regression models were fitted accordingly. Interaction effects were assessed using likelihood ratio tests. A *p-*value of >0.05 indicated no significant interaction, whereas a *p-*value of ≤0.05 suggested possible effect modification. Additionally, generalized additive models with smoothing splines were employed to examine the dose–response relationship between METS-IR and GDM.

Finally, receiver operating characteristic (ROC) curves were used to evaluate the predictive performance of METS-IR for GDM, preterm birth, macrosomia, GDM&PE, and GDMA2. The area under the curve (AUC) and optimal cut-off values were calculated to quantify the discriminative ability of METS-IR.

All statistical analyses were performed using Statistical Package for the Social Sciences (SPSS) software (version 21.0, IBM Corporation, Armonk, NY, USA) and R software (version 4.4.1, R Foundation for Statistical Computing). Two-sided *p*-values of <0.05 were considered statistically significant.

## Results

### Baseline characteristics

Table 1 presents the baseline characteristics of participants stratified by METS-IR quartiles. A total of 37,770 participants who met the inclusion and exclusion criteria were evenly distributed across the four groups. Analysis showed that only smoking history and alcohol consumption differed significantly across groups (*p* > 0.05), while variables such as age, prepregnancy BMI, and gestational week at METS-IR measurement did not show significant differences among the four groups (*p* < 0.05). The prevalence of GDM varied from 8.70 to 21.54% across METS-IR quartiles. Compared with the lowest METS-IR group, the higher METS-IR groups exhibited a significantly higher incidence of GDM. In addition, the incidence of macrosomia, GDM&PE, and GDMA2 was also markedly higher in the high METS-IR groups.

**Table 1:** Baseline characteristics of participants.

Characteristic	METS-IR tertile				
	Q1 (21.58, 32.18)	Q2 (32.18, 36.10)	Q3 (36.10, 40.91)	Q4 (40.91, 58.43)	*p*-value
Participants	9,443	9,442	9,442	9,443	
Age (years)	30.76 ± 3.87	31.25 ± 3.93	31.49 ± 4.09	31.71 ± 4.22	<0.001
BMI (kg/m^2^)	18.73 ± 1.29	20.35 ± 1.44	21.71 ± 1.75	24.59 ± 2.66	<0.001
METS-IR test week	11.00 ± 2.49	10.87 ± 2.44	10.96 ± 2.45	10.95 ± 2.51	<0.001
METS-IR	29.57 ± 1.92	34.11 ± 1.13	38.32 ± 1.37	45.90 ± 4.16	<0.001
TG (mmol/L)	1.10 ± 0.41	1.23 ± 0.48	1.37 ± 0.57	1.61 ± 0.80	<0.001
HDL (mmol/L)	1.89 ± 0.34	1.60 ± 0.36	1.40 ± 0.36	1.23 ± 0.33	<0.001
FPG (mmol/L)	4.41 ± 0.38	4.47 ± 0.39	4.52 ± 0.43	4.62 ± 0.51	<0.001
Cr (U/L)	43.03 ± 5.87	43.17 ± 5.88	43.49 ± 6.02	44.02 ± 6.11	<0.001
ALT (U/L)	16.02 ± 13.12	17.19 ± 15.36	18.47 ± 16.17	20.79 ± 17.95	<0.001
TC (mmol/L)	4.63 ± 0.79	4.56 ± 0.78	4.54 ± 0.78	4.47 ± 0.80	<0.001
Aspirin (%)					<0.001
No	9,576 (98.67%)	9,561 (98.51%)	9,549 (98.40%)	9,483 (97.69%)	
Yes	129 (1.33%)	145 (1.49%)	155 (1.60%)	224 (2.31%)	
Hypertension history (%)					<0.001
No	8,213 (86.98%)	8,148 (86.30%)	8,089 (85.69%)	7,906 (83.75%)	
Yes	1,229 (13.02%)	1,293 (13.70%)	1,351 (14.31%)	1,534 (16.25%)	
Diabetes history					<0.001
No	8,985 (95.16%)	8,966 (94.97%)	8,856 (93.81%)	8,743 (92.62%)	
Yes	457 (4.84%)	475 (5.03%)	584 (6.19%)	697 (7.38%)	
Tobacco (%)					0.49
No	8,703 (98.85%)	8,725 (98.61%)	8,794 (98.73%)	8,822 (98.65%)	
Yes	101 (1.15%)	123 (1.39%)	113 (1.27%)	121 (1.35%)	
Alcohol (%)					0.06
No	8,532 (96.91%)	8,566 (96.81%)	8,640 (97.00%)	8,715 (97.45%)	
Yes	272 (3.09%)	282 (3.19%)	267 (3.00%)	228 (2.55%)	
Parity (%)					<0.001
Primipara	7,516 (79.59%)	7,079 (74.97%)	6,822 (72.25%)	6,739 (71.37%)	
Multipara	1,927 (20.41%)	2,363 (25.03%)	2,620 (27.75%)	2,704 (28.63%)	
IVF (%)					0.004
No	8,958 (94.86%)	8,890 (94.15%)	8,880 (94.05%)	8,844 (93.66%)	
Yes	485 (5.14%)	552 (5.85%)	562 (5.95%)	599 (6.34%)	
Adverse pregnancy history (%)					<0.001
No	9,090 (96.26%)	9,042 (95.76%)	8,987 (95.18%)	8,997 (95.28%)	
Yes	353 (3.74%)	400 (4.24%)	455 (4.82%)	446 (4.72%)	
Education (%)					<0.001
Postgraduate	2,416 (27.14%)	2,245 (25.22%)	1,885 (21.31%)	1,414 (16.15%)	
Bachelor’s degree or above	4,168 (46.82%)	4,157 (46.69%)	4,261 (48.18%)	4,043 (46.18%)	
College diploma	1,536 (17.25%)	1,663 (18.68%)	1,824 (20.62%)	2,259 (25.81%)	
High school	227 (2.55%)	243 (2.73%)	282 (3.19%)	365 (4.17%)	
Less than junior high school	556 (6.25%)	595 (6.68%)	592 (6.69%)	673 (7.69%)	
GDM					<0.001
No	8,621 (91.30%)	8,453 (89.53%)	8,121 (86.01%)	7,409 (78.46%)	
Yes	822 (8.70%)	989 (10.47%)	1,321 (13.99%)	2034 (21.54%)	
Preterm birth					<0.001
No	8,991 (95.22%)	8,988 (95.19%)	8,985 (95.16%)	8,854 (93.76%)	
Yes	451 (4.78%)	454 (4.81%)	457 (4.84%)	589 (6.24%)	
Macrosomia					<0.001
No	8,996 (95.60%)	8,802 (93.52%)	8,485 (90.13%)	8,033 (85.36%)	
Yes	414 (4.40%)	610 (6.48%)	929 (9.87%)	1,378 (14.64%)	
GDM&PE					<0.001
No	9,403 (99.58%)	9,401 (99.57%)	9,369 (99.23%)	9,184 (97.26%)	
Yes	40 (0.42%)	41 (0.43%)	73 (0.77%)	259 (2.74%)	
GDMA2					<0.001
No	4,912 (99.19%)	5,636 (98.76%)	6,533 (97.78%)	7,055 (94.95%)	
Yes	40 (0.81%)	71 (1.24%)	148 (2.22%)	375 (5.05%)	

Mean ± SD for continuous variables: *p*-value was calculated by a weighted linear regression model.

% for categorical variables: *p*-value was calculated by a weighted chi-squared test.

METS-IR, metabolic score for insulin resistance; Q, quartile; BMI, pre-pregnancy body mass index; TG, triglycerides; HDL, high-density lipoprotein; FPG, fasting plasma glucose; Cr, Creatinine; ALT, alanine aminotransferase; TC, total cholesterol; IVF, in vitro fertilization; GDM, gestational diabetes mellitus; GDM&PE, gestational diabetes mellitus with preeclampsia; GDMA2, gestational diabetes mellitus managed with insulin therapy.

### Association between METS-IR and GDM

Table 2 summarizes the associations between METS-IR and the primary outcome (GDM) and secondary outcomes (preterm birth, macrosomia, GDM&PE, and GDMA2), stratified by METS-IR quartiles, with the lowest quartile (Q1) serving as the reference group. In model I (unadjusted), the multivariable regression analysis revealed that, compared with Q1, participants in the higher METS-IR groups had significantly increased risks of GDM, macrosomia, and GDMA2 (*p* < 0.05). However, a significant increase in risk for preterm birth and GDM&PE was observed only in the highest quartile (Q4) (*p* < 0.05), with no significant differences in Q2 and Q3.

**Table 2:** The associations between METS-IR and risk of primary and secondary outcomes.

Outcome	Number (%)	Model I		Model II	
		OR (95% CI)	*p*-value	Adjust OR (95% CI)	*p*-value
GDM					
Q1	822 (8.70%)	Reference		Reference	
Q2	989 (10.47%)	1.23 (1.11, 1.35)	<0.0001	1.13 (1.02, 1.25)	0.0199
Q3	1,321 (13.99%)	1.71 (1.56, 1.87)	<0.0001	1.59 (1.44, 1.75)	<0.0001
Q4	2,034 (21.54%)	2.88 (2.64, 3.14)	<0.0001	2.65 (2.42, 2.91)	<0.0001
Preterm birth					
Q1	451 (4.78%)	Reference		Reference	
Q2	454 (4.81%)	1.01 (0.88, 1.15)	0.9186	0.97 (0.84, 1.11)	0.6245
Q3	457 (4.84%)	1.01 (0.89, 1.16)	0.8383	0.97 (0.84, 1.12)	0.7088
Q4	589 (6.24%)	1.33 (1.17, 1.50)	<0.0001	1.21 (1.06, 1.39)	0.0061
Macrosomia					
Q1	414 (4.40%)	Reference		Reference	
Q2	610 (6.48%)	1.51 (1.32, 1.71)	<0.0001	1.51 (1.32, 1.73)	<0.0001
Q3	929 (9.87%)	2.38 (2.11, 2.68)	<0.0001	2.36 (2.09, 2.68)	<0.0001
Q4	1,378 (14.64%)	3.73 (3.33, 4.18)	<0.0001	3.72 (3.30, 4.20)	<0.0001
GDM&PE					
Q1	40 (0.42%)	Reference		Reference	
Q2	41 (0.43%)	1.03 (0.66, 1.59)	0.9110	1.02 (0.65, 1.60)	0.9235
Q3	73 (0.77%)	1.83 (1.24, 2.70)	0.0022	1.71 (1.15, 2.56)	0.0087
Q4	259 (2.74%)	6.63 (4.75, 9.26)	<0.0001	5.99 (4.22, 8.50)	<0.0001
GDMA2					
Q1	40 (0.81%)	Reference		Reference	
Q2	71 (1.24%)	1.55 (1.05, 2.28)	0.0281	1.40 (0.94, 2.08)	0.0964
Q3	148 (2.22%)	2.78 (1.96, 3.95)	<0.0001	2.51 (1.76, 3.58)	<0.0001
Q4	375 (5.05%)	6.53 (4.70, 9.06)	<0.0001	5.58 (4.00, 7.79)	<0.0001

Model I: No covariates were adjusted.

Model II: Adjusted for age, test week, Cr, ALT, TC, hypertension history, diabetes history, tobacco, alcohol, IVF, adverse pregnancy history, parity, and education levels.

METS-IR, metabolic score for insulin resistance; Q, quartile; OR, odds ratio; CI, confidence interval; GDM, gestational diabetes mellitus; GDM&PE, gestational diabetes mellitus with preeclampsia; GDMA2, gestational diabetes mellitus managed with insulin therapy.

In model II (adjusted for potential confounders), all three higher METS-IR groups showed statistically significant increases in GDM risk compared with Q1. Specifically, the risk of GDM increased by 13% in Q2 (OR = 1.13, 95% CI: 1.02–1.25), 59% in Q3 (OR = 1.59, 95% CI: 1.44–1.75), and 165% in Q4 (OR = 2.65, 95% CI: 2.42–2.91).

The risk of preterm birth did not significantly increase in Q2 and Q3 but increased by 21% in Q4 (OR = 1.21, 95% CI: 1.06–1.39). The risk of macrosomia closely mirrored that of GDM, showing significant increases in all higher METS-IR groups, especially in Q4, where the risk increased by 272% (OR = 3.72, 95% CI: 3.30–4.20). The risk of GDM&PE was significantly elevated in Q3 and Q4, with a 499% increase in Q4 (OR = 5.99, 95% CI: 4.22–8.50). Similarly, the risk of GDMA2 increased significantly in Q3 and Q4, with a 458% increase in Q4 (OR = 5.58, 95% CI: 4.00–7.79).

**Figure 2:**
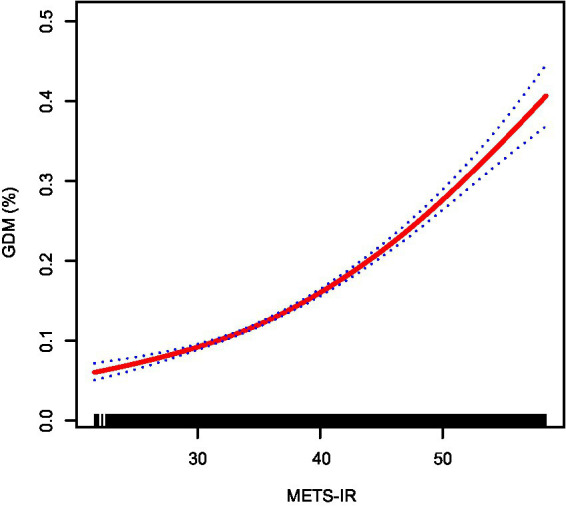
Smooth curve fitting showing the nonlinear relationship between METS-IR and the risk of GDM. The red solid line represents the probability of GDM occurrence, and the blue dotted line indicates the 95% confidence interval (CI) curve. METS-IR, metabolic score for insulin resistance; GDM, gestational diabetes mellitus.

Furthermore, generalized additive models with smoothing splines suggested a near-linear dose–response relationship between METS-IR levels and GDM risk (Figure 2), indicating that higher METS-IR values were consistently associated with increased GDM risk.

### Sensitivity analysis

**Figure 3:**
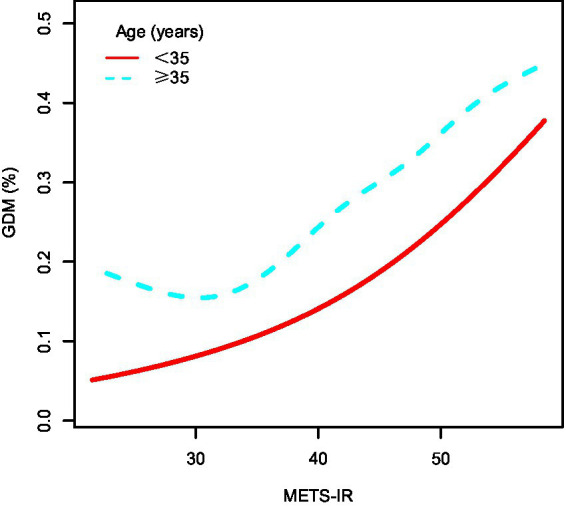
The dose–response relationship between METS-IR and GDM, stratified by age. METS-IR, metabolic score for insulin resistance; GDM, gestational diabetes mellitus.

To further explore the association between METS-IR and GDM, preterm birth, macrosomia, GDM&PE, and GDMA2, subgroup analyses were conducted based on maternal age. Table 3 presents the results of these subgroup analyses. Within the same METS-IR quartile, the incidence of GDM was clearly higher among women aged ≥ 35 years compared to those younger than 35 years (Q1: 7.60% vs. 15.37%; Q2: 9.31% vs. 16.31%; Q3: 12.06% vs. 22.05%; Q4: 19.11% vs. 30.35%). Figure 3 illustrates the dose–response relationship between METS-IR and GDM risk derived from smoothed curve fitting. The results indicated that, at the same METS-IR level, women aged ≥35 years had a higher risk of GDM, and the overall trend of increasing GDM risk with rising METS-IR was consistent across age groups.

**Table 3:** Subgroup analysis of the METS-IR index and primary and secondary outcomes.

Subgroup	Number of Participants (%)	Non-adjusted OR (95% CI)	*p*-value	*p*-value for interaction	Adjusted OR (95% CI)	*p*-value	*p*-value for interaction
GDM				0.341			0.5156
Age < 35 years							
Q1	630 (7.69%)	Reference			Reference		
Q2	733 (9.31%)	1.23 (1.10, 1.38)	0.0002		1.16 (1.03, 1.30)	0.0138	
Q3	918 (12.06%)	1.65 (1.48, 1.83)	<0.0001		1.60 (1.43, 1.79)	<0.0001	
Q4	1,416 (19.11%)	2.84 (2.57, 3.13)	<0.0001		2.74 (2.46, 3.05)	<0.0001	
Age ≥ 35 years							
Q1	192 (15.37%)	Reference			Reference		
Q2	256 (16.31%)	1.07 (0.87, 1.32)	0.5008		1.04 (0.84, 1.28)	0.7352	
Q3	403 (22.05%)	1.56 (1.29, 1.88)	<0.0001		1.52 (1.25, 1.85)	<0.0001	
Q4	618 (30.35%)	2.40 (2.00, 2.87)	<0.0001		2.36 (1.95, 2.85)	<0.0001	
Preterm birth				0.995			0.9737
Age < 35 years							
Q1	371 (4.53%)	Reference			Reference		
Q2	352 (4.47%)	0.99 (0.85, 1.15)	0.8624		0.97 (0.83, 1.14)	0.6987	
Q3	340 (4.47%)	0.99 (0.85, 1.15)	0.8504		0.96 (0.82, 1.13)	0.6352	
Q4	426 (5.75%)	1.29 (1.11, 1.48)	0.0006		1.22 (1.05, 1.43)	0.0109	
Age ≥ 35 years							
Q1	80 (6.41%)	Reference			Reference		
Q2	102 (6.50%)	1.02 (0.75, 1.37)	0.9216		0.95 (0.69, 1.31)	0.7695	
Q3	117 (6.40%)	1.00 (0.74, 1.34)	0.9958		0.98 (0.73, 1.33)	0.9204	
Q4	163 (8.02%)	1.27 (0.97, 1.68)	0.0873		1.17 (0.87, 1.56)	0.3048	
Macrosomia				0.0795			0.1236
Age < 35 years							
Q1 =	341 (4.18%)	Reference			Reference		
Q2	497 (6.33%)	1.55 (1.35, 1.79)	<0.0001		1.57 (1.36, 1.82)	<0.0001	
Q3	716 (9.43%)	2.39 (2.09, 2.73)	<0.0001		2.39 (2.08, 2.74)	<0.0001	
Q4	1,077 (14.58%)	3.92 (3.45, 4.44)	<0.0001		3.95 (3.45, 4.51)	<0.0001	
Age ≥ 35 years							
Q1	73 (5.86%)	Reference			Reference		
Q2	113 (7.23%)	1.25 (0.92, 1.69)	0.1501		1.26 (0.92, 1.73)	0.1431	
Q3	213 (11.69%)	2.13 (1.61, 2.80)	<0.0001		2.20 (1.65, 2.92)	<0.0001	
Q4	301 (14.87%)	2.80 (2.15, 3.66)	<0.0001		2.90 (2.20, 3.83)	<0.0001	
GDM&PE				0.4601			0.6104
Age < 35 years							
Q1	30 (0.37%)	Reference			Reference		
Q2	32 (0.41%)	1.11 (0.67, 1.83)	0.6800		1.08 (0.64, 1.80)	0.7805	
Q3	44 (0.58%)	1.58 (0.99, 2.52)	0.0533		1.51 (0.94, 2.44)	0.0904	
Q4	168 (2.27%)	6.31 (4.28, 9.32)	<0.0001		5.72 (3.82, 8.56)	<0.0001	
Age ≥ 35 years							
Q1	10 (0.80%)	Reference			Reference		
Q2	9 (0.57%)	0.71 (0.29, 1.76)	0.4655		0.87 (0.34, 2.20)	0.7658	
Q3	29 (1.59%)	2.00 (0.97, 4.11)	0.0605		2.17 (1.01, 4.66)	0.0471	
Q4	91 (4.48%)	5.81 (3.01, 11.20)	<0.0001		6.32 (3.13, 12.77)	<0.0001	
GDMA2				0.9731			0.9958
Age < 35 years							
Q1	29 (0.68%)	Reference			Reference		
Q2	49 (1.02%)	1.51 (0.95, 2.40)	0.0789		1.41 (0.88, 2.25)	0.1498	
Q3	97 (1.79%)	2.67 (1.76, 4.05)	<0.0001		2.53 (1.66, 3.85)	<0.0001	
Q4	239 (4.10%)	6.26 (4.25, 9.23)	<0.0001		5.49 (3.71, 8.14)	<0.0001	
Age ≥ 35 years							
Q1	11 (1.63%)	Reference			Reference		
Q2	22 (2.42%)	1.49 (0.72, 3.10)	0.2812		1.34 (0.63, 2.83)	0.4480	
Q3	51 (4.04%)	2.53 (1.31, 4.90)	0.0056		2.47 (1.27, 4.81)	0.0079	
Q4	136 (8.51%)	5.60 (3.01, 10.42)	<0.0001		5.56 (2.95, 10.47)	<0.0001	

Non-adjusted model had no adjustments. Each stratification was adjusted for age, test week, Cr, ALT, TC, hypertension history, diabetes history, tobacco, alcohol, IVF, adverse pregnancy history, parity, and education levels. METS-IR, metabolic score for insulin resistance; Q, quartile; OR, odds ratio; CI, confidence interval; GDM, gestational diabetes mellitus; GDM&PE, gestational diabetes mellitus with preeclampsia; GDMA2, gestational diabetes mellitus managed with insulin therapy.

After adjusting for confounding factors, the association between METS-IR and GDM remained consistent across all quartiles in women younger than 35 years. Compared with Q1, the risk of GDM increased by 52% in Q3 (OR = 1.52, 95% CI: 1.25–1.85) and by 136% in Q4 (OR = 2.36, 95% CI: 1.95–2.85). Subgroup analysis for macrosomia yielded findings similar to those for GDM. The associations between METS-IR and GDM&PE or GDMA2 were generally consistent across age groups. For preterm birth, after adjustment, a significant increase in risk was observed in Q4 among women younger than 35 years (OR = 1.22, 95% CI: 1.05–1.43). However, no significant differences were found among different METS-IR groups in women aged ≥ 35 years. The interaction test showed no statistically significant differences among all the subgroups (interaction *p* > 0.05).

### ROC analysis

**Figure 4:**
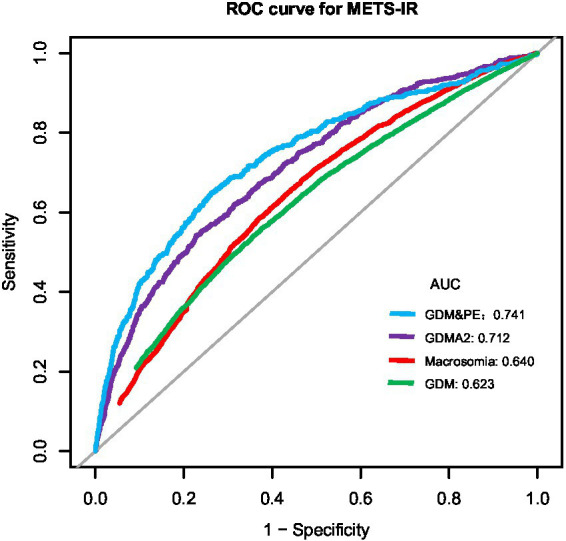
ROC curves for METS-IR to predict the risk of GDM, preterm birth, macrosomia, GDM&PE, and GDMA2 in all participants. ROC, receiver operating characteristic; AUC, area under the curve; METS-IR, metabolic score for insulin resistance; GDM, gestational diabetes mellitus; GDM&PE, GDM complicated with preeclampsia; GDMA2, pharmacologically treated GDM.

ROC analysis was used to evaluate the predictive performance of METS-IR for GDM and its related adverse outcomes. As shown in Table 4 and Figure 4, the AUC for GDM, preterm birth, macrosomia, GDM&PE, and GDMA2 was 0.623 (95% CI: 0.614–0.631), 0.532 (95% CI: 0.518–0.546), 0.640 (95% CI: 0.631–0.650), 0.741 (95% CI: 0.715–0.767), and 0.712 (95% CI: 0.691–0.733), respectively. Using the Youden index, optimal cut-off values for predicting GDM, preterm birth, macrosomia, GDM&PE, and GDMA2 were identified as 38.118, 36.633, 37.992, 40.509, and 42.314, respectively. The corresponding specificities were 64.4, 65.0, 63.1, 73.8, and 77.2%, respectively, and the sensitivities were 54.0, 41.3, 58.4, 64.9, and 54.3%, respectively.

**Table 4:** Results of ROC analysis of the METS-IR index used to predict the development of primary and secondary outcomes.

Outcomes	AUC (95% CI)	Sensitivity	Specificity	PPV	NPV	Best threshold
GDM	0.623 (0.614, 0.631)	0.540	0.644	0.194	0.898	38.118
Preterm birth	0.532 (0.518, 0.546)	0.413	0.650	0.060	0.953	38.633
Macrosomia	0.640 (0.631, 0.650)	0.584	0.631	0.133	0.940	37.992
GDM&PE	0.741 (0.715, 0.767)	0.649	0.738	0.027	0.995	40.509
GDMA2	0.712 (0.691, 0.733)	0.543	0.772	0.059	0.985	42.314

ROC, receiver operating characteristic; AUC, area under the curve; METS-IR, metabolic score for insulin resistance; PPV, positive *p*-value; NPV, negative *p*-value; GDM, gestational diabetes mellitus; GDM&PE, gestational diabetes mellitus with preeclampsia; GDMA2, gestational diabetes mellitus managed with insulin therapy.

## Discussion

In this study, we conducted a retrospective cohort analysis of 37,770 pregnant women from three hospitals in China, aiming to investigate the association between METS-IR and GDM, as well as its related adverse pregnancy outcomes. After adjusting for potential confounding factors, the results demonstrated a positive correlation between METS-IR and the risk of GDM. Subgroup analyses stratified by maternal age confirmed a consistent positive association between METS-IR and GDM. Moreover, METS-IR exhibited good discriminative ability in predicting GDM and its complications. Notably, it showed strong predictive performance for GDM&PE and GDMA2, with AUC values of 0.741 (95% CI: 0.715–0.767) and 0.712 (95% CI: 0.691–0.733), respectively. These findings indicate that METS-IR, as a simple and non-invasive metabolic assessment tool, holds potential clinical value in risk stratification and severity prediction of GDM.

Previous studies have established that increased IR is a central mechanism in the development of GDM (24). The EHC technique is considered the gold standard for assessing insulin resistance. However, it is complex, costly, and invasive, making it unsuitable for routine clinical use. As a result, several surrogate indices, including the Quantitative Insulin Sensitivity Check Index (QUICKI), the homeostasis model assessment of insulin resistance (HOMA-IR), the triglyceride-glucose (TyG) index, and the TG/HDL-C, have been used to evaluate insulin resistance and its association with GDM (25, 26). However, the clinical validity of these indices remains uncertain. METS-IR is non-invasive and easily calculated from routine clinical data. Evidence has shown that METS-IR demonstrates better diagnostic performance for insulin resistance compared to EHC (11), and it has been identified as an independent predictor of type 2 diabetes mellitus (13). Our findings indicate that, as METS-IR percentile scores increase, so does the risk of GDM in a graded manner. Compared with the lowest quartile, individuals in the highest METS-IR quartile had a 165% higher risk of developing GDM. This observation aligns with previous findings in general populations. For instance, Cheng et al. (14) reported a significant association between elevated METS-IR and T2DM incidence (OR: 1.804; 95% CI: 1.720–1.891). Another cross-sectional study conducted in China provided similar evidence of a positive correlation (27), and a subsequent 6-year longitudinal study found that each one-standard-deviation increase in METS-IR was associated with an 82% higher risk of developing diabetes (28). More recently, a cohort study in a Japanese population showed that participants in the highest METS-IR quartile had a 215% higher risk of developing diabetes compared to those in the lowest quartile (13). Recent studies have reported a positive association between elevated METS-IR and an increased risk of GDM (16, 17). Building on these findings, our multicenter study with a large sample size, rigorous control of confounders, and comprehensive statistical analyses further confirms the association between METS-IR and GDM. Given the practicality of METS-IR measurement and its strong pathophysiological link to IR, its application in identifying high-risk individuals for GDM appears feasible. To further assess the predictive performance of METS-IR for GDM, we conducted ROC curve analysis, which yielded an AUC of 0.623 (95% CI: 0.614–0.631), with a specificity of 64.4% and a sensitivity of 54.0%. Although the AUC is less than 0.7, METS-IR may be considered as a potential indicator for GDM risk stratification, helping to identify individuals at higher risk during early pregnancy.

In this study, we further evaluated the association between METS-IR and multiple adverse pregnancy outcomes related to GDM. The results showed no significant association between METS-IR and preterm birth, whereas significant positive correlations were observed between METS-IR and macrosomia, GDM&PE, and GDMA2. These findings suggest that METS-IR not only reflects overall IR in pregnant women but may also uncover underlying pathophysiological mechanisms of GDM-related complications. Emerging evidence has demonstrated that IR is closely linked to both GDM and its adverse perinatal outcomes. For instance, compared with women with normal glucose tolerance, those with GDM exhibit higher levels of IR accompanied by more severe metabolic disturbances and worse perinatal outcomes (29). Moreover, insulin resistance has been recognized as one of the key metabolic underpinnings of preeclampsia (PE) (30). However, unlike some previous studies that reported a significant association between IR and preterm birth (8), our study did not detect a statistically significant relationship between METS-IR and preterm birth. This inconsistency may stem from differences in calculation methods and clinical applicability among various IR assessment tools, or it could be due to variations in population characteristics and the extent of confounding adjustment across studies. Notably, our findings revealed that METS-IR demonstrated good predictive performance for GDM&PE and GDMA2, with AUC values of 0.741 and 0.712, respectively, exceeding the performance of several traditional risk parameters. IR not only represents metabolic dysregulation but is also closely associated with endothelial dysfunction (31), and these shared mechanisms may form the basis for the co-occurrence of GDM and PE. Furthermore, accumulating evidence suggests that multiple pathological pregnancy conditions, including PE, GDM, and obesity, are characterized by reduced insulin signaling in the fetal-placental vasculature (32, 33). Therefore, as a surrogate marker of insulin resistance, METS-IR holds clinical value not only in identifying high-risk individuals for GDM but also in predicting severe GDM subtypes complicated by preeclampsia or requiring insulin therapy.

Given that advanced maternal age (≥35 years) is a well-established independent risk factor for GDM (34), we conducted a subgroup analysis stratified by age. The results were consistent with expectations, showing a significantly higher incidence of GDM in the ≥ 35 years group. The positive association between METS-IR and GDM was largely consistent across age groups, indicating that METS-IR is a feasible and stable tool for risk stratification in pregnant women of different ages. However, within the ≥ 35 years subgroup, a significant association between METS-IR and GDM was observed only in Q3 and Q4, but not in Q2. To explain this discrepancy, we propose the following possibilities: It has been documented that, with increasing age, pancreatic β-cell function declines, insulin sensitivity decreases, and glucose metabolism becomes more impaired, thereby elevating the risk of GDM (35, 36). Nevertheless, some older pregnant women may already have a certain degree of insulin resistance prior to pregnancy, which could potentially reduce the sensitivity of METS-IR in capturing changes in IR levels. Additionally, age is an important determinant of lipid metabolism. Studies have shown that HDL-C levels tend to decline with advancing age (37). Since both FPG and HDL-C are key components in the calculation of METS-IR, their age-related variations may affect the stability of the score and consequently reduce its accuracy in predicting GDM. Finally, as an independent risk factor for GDM, age may contribute to disease development through mechanisms not directly related to IR. Therefore, in clinical practice, it is essential to integrate other risk factors alongside METS-IR when assessing GDM risk in older pregnant women.

The association between METS-IR and GDM may involve multiple interacting pathophysiological mechanisms. IR is a central mechanism in the development of GDM. During normal pregnancy, a progressive increase in IR serves as an adaptive response to meet the growing energy demands of both mother and fetus (38). However, when IR becomes excessively elevated, it can lead to inadequate β-cell compensation, resulting in glucose metabolic imbalance and increased risk of GDM (39, 40). As a composite indicator of insulin resistance, elevated METS-IR reflects increased IR and thus holds potential value in identifying individuals at higher risk. Dyslipidemia also plays a significant role in GDM progression. Studies have shown that, compared with women with normal pregnancies, those with GDM often exhibit lower HDL-C levels and higher levels of TG, TC, and LDL-C (41, 42). Since METS-IR incorporates both TG and HDL-C into its scoring system, it partially reflects the degree of lipid metabolic disturbance. Chronic inflammation is another key link connecting insulin resistance and GDM. Pro-inflammatory cytokines such as IL-6 can suppress lipoprotein lipase activity, promote TG accumulation, and exacerbate IR (43). In addition, inflammatory mediators, such as IL-1β, can activate multiple signaling pathways and directly impair pancreatic β-cell function (44, 45). C-reactive protein (CRP) further contributes to systemic inflammation and worsens insulin action defects (46). Previous studies have demonstrated a positive correlation between METS-IR and inflammatory markers, such as CRP and IL-6 (47). Oxidative stress also contributes to insulin resistance by activating the NF-κB pathway, leading to endothelial dysfunction and impaired IR (48). Moreover, lifestyle factors such as dietary patterns and physical activity levels significantly influence insulin sensitivity and thereby modulate the risk of developing GDM. In summary, the relationship between METS-IR and GDM likely results from the complex interplay of multiple metabolic, inflammatory, and oxidative stress mechanisms.

The main strengths of this study lie in its multicenter design and large sample size, which effectively reduced bias caused by small sample sizes and enhanced statistical power, thereby improving the generalizability and applicability of the findings. Moreover, METS-IR is a simple, non-invasive, and easily obtainable metabolic index with good clinical feasibility, making it suitable for widespread application in routine clinical practice and highlighting its important clinical value. Despite these strengths, several limitations should be acknowledged. First, this study was based only on baseline measurements before 24 weeks of gestation. Since METS-IR may vary dynamically across gestation, future prospective studies with multiple time points are needed to explore longitudinal changes in METS-IR and their association with GDM. Second, all participants were from China. Given potential differences in genetic background, lifestyle, and metabolic profiles across populations, future studies in multiethnic and multicenter settings are necessary to confirm the generalizability of our findings. Finally, as a retrospective cohort study, our analysis excluded some patients due to incomplete clinical data. Additionally, information on exercise, dietary interventions, and insulin use was not collected. Although we adjusted for multiple confounders, potential selection bias may still influence the results.

## Conclusion

Our findings demonstrate that the METS-IR score is positively associated with the risk of GDM and has a certain predictive value for GDM occurrence, particularly in identifying severe subtypes such as GDM&PE or GDMA2. As a novel, simple, and easily accessible marker of insulin resistance, METS-IR holds promise for use in risk stratification and early intervention strategies for GDM, offering valuable clinical insights with strong practical implications.

## Data Availability

The original contributions presented in the study are included in the article/supplementary material; further inquiries can be directed to the corresponding author/s.

## Glossary

GDM: Gestational diabetes mellitus
IR: Insulin resistance
EHC: Euglycemic-hyperinsulinemic clamp
METS-IR: Metabolic score for insulin resistance
FPG: Fasting plasma glucose
TG: Triglycerides
HDL-C: High-density lipoprotein cholesterol
BMI: Body mass index
ALT: Alanine aminotransferase
Cr: Creatinine
TC: Total cholesterol
IVF: *In vitro* fertilization
OGTT: Oral glucose tolerance test
GDM&PE: GDM complicated with preeclampsia
GDMA2: Pharmacologically treated GDM
Q: Quartile
SD: Standard deviation
OR: Odds ratio
CI: Confidence intervals
ROC: Receiver operating characteristic
AUC: Area under the curve
